# Role and Mechanism of Arsenic in Regulating Angiogenesis

**DOI:** 10.1371/journal.pone.0020858

**Published:** 2011-06-08

**Authors:** Ling-Zhi Liu, Yue Jiang, Richard L. Carpenter, Yi Jing, Stephen C. Peiper, Bing-Hua Jiang

**Affiliations:** Department of Pathology, Anatomy and Cell Biology, Thomas Jefferson University, Philadelphia, Pennsylvania, United States of America; The University of Kansas Medical Center, United States of America

## Abstract

Arsenic is a wide spread carcinogen associated with several kinds of cancers including skin, lung, bladder, and liver cancers. Lung is one of the major targets of arsenic exposure. Angiogenesis is the pivotal process during carcinogenesis and chronic pulmonary diseases, but the role and mechanism of arsenic in regulating angiogenesis remain to be elucidated. In this study we show that short time exposure of arsenic induces angiogenesis in both human immortalized lung epithelial cells BEAS-2B and adenocarcinoma cells A549. To study the molecular mechanism of arsenic-inducing angiogenesis, we find that arsenic induces reactive oxygen species (ROS) generation, which activates AKT and ERK1/2 signaling pathways and increases the expression of hypoxia-inducible factor 1 (HIF-1) and vascular endothelial growth factor (VEGF). Inhibition of ROS production suppresses angiogenesis by decreasing AKT and ERK activation and HIF-1 expression. Inhibition of ROS, AKT and ERK1/2 signaling pathways is sufficient to attenuate arsenic-inducing angiogenesis. HIF-1 and VEGF are downstream effectors of AKT and ERK1/2 that are required for arsenic-inducing angiogenesis. These results shed light on the mechanism of arsenic in regulating angiogenesis, and are helpful to develop mechanism-based intervention to prevent arsenic-induced carcinogenesis and angiogenesis in the future.

## Introduction

Arsenic is a well-documented carcinogen associated with human lung, skin, urinary bladder, kidney and liver cancers [1]–[3]. Arsenic causes DNA-strand break, loss of DNA methylation, cellular endocrine disorder and cell transformation [4]–[9]. Arsenic also induces oxidative stress, genotoxic damage, DNA repair inhibition, epigenetic events and activation of certain signal transduction pathways leading to aberrant gene expression [10]. The WHO sets the maximum safe level of arsenic at 0.01 mg/L, while arsenic levels in water in some areas can be up to several thousand times higher than the limit [11]. The lung cancer is highly associated with arsenic exposure [1], [12], [13]. In addition to lung cancer, arsenic exposure also leads to other cancers and non-malignant pulmonary diseases including chronic bronchitis and chronic obstructive pulmonary diseases (COPD) [14]. However, the mechanisms of arsenic exposure leading to the lung carcinogenesis and other pulmonary diseases remain to be elucidated.

Angiogenesis is the process that new capillaries are generated from the pre-existing vasculature which plays a pivotal role in the initiation of carcinogenesis and tumor progress, vascular diseases, and various ischemic and inflammatory diseases [15], [16]. Tumor cannot grow without angiogenesis when it reaches 1∼2 mm in diameter [17]. Angiogenesis is also a prominent feature of the structural tissue remodeling that occurs in the chronic airway diseases including COPD, in which the angiogenesis/angiostatic balance is affected [18], [19]. VEGF is the most potent angiogenic factor. Our previous studies demonstrated that arsenic induced VEGF expression in human prostate cancer cells and tumor angiogenesis [20], [21]. Acute arsenic treatment also induced VEGF expression in human microvascular endothelial cells through the induction of heme oxygenase-1 gene expression [22]. It has been reported that sodium arsenite (arsenic) treatment induced angiogenesis responses using the chick chorioallantoic membrane (CAM) model [23]. Recent study showed that the chronic exposure of arsenic in drinking water caused both liver angiogenesis and pathogenic liver sinusoidal endothelial cell capillarization in mice mediated by sphingosine-1-phosphate type 1 receptor in endothelial cells [24]. However, it is unknown whether acute arsenic exposure may affect epithelial cells to induce angiogenesis *in vivo*.

In this study, we used human immortalized normal lung epithelial cells BEAS-2B to determine: 1) whether short time exposure of arsenic induces angiogenesis; 2) what signaling pathways are involved in arsenic-inducing angiogenesis; 3) whether VEGF and HIF-1 are required for arsenic-inducing angiogenesis. Given the important role of angiogenesis in lung cancer and other arsenic-induced pulmonary diseases, these results may shed light on the mechanism of arsenic-induced angiogenesis and help to develop mechanism-based interventions to prevent carcinogenesis and other diseases induced by arsenic in the future.

## Materials and Methods

### Cell Culture

Human immortalized lung epithelial cells, BEAS-2B cells (ATCC, Manassas, VA) were cultured in DMEM medium supplemented with 10% fetal bovine serum (FBS) and 100 units/ml penicillin, and 100 µg/ml streptomycin, and cultured at 37°C in a 5% CO_2_ incubator. Human lung carcinoma cells, A549 cells (ATCC, Manassas, VA) were maintained in RPMI 1640 medium with 10% FBS and antibiotics as above.

### Angiogenesis Assay

Fertilized chicken eggs (Charles River Laboratories International, Inc., Wilmington, MA) were incubated at 37°C for 9 days, and angiogenesis assay was performed as we previously described [25], [26]. In brief, BEAS-2B cells and A549 cells were treated with 5 µM of sodium arsenic (Sigma, St. Louis, MO) for 5 h. Then cells were trypsinized, resuspended, mixed with Matrigel, without or with LY294002 (PI3K inhibitor) and U0126 (MAPK/ERK inhibitor), and implanted onto the CAM at Day 9. Adenoviruses carrying GFP, AKT-DN, HIF-1α siRNA (si HIF-1α), and catalase were used to infect BEAS-2B cells at 30 MOI for 24 h before the arsenic treatment. Similarly, BEAS-2B cells were transfected by siRNA oligomers of siScrambled control (siSCR), MAPK siRNA (siMAPK), and VEGF (siVEGF) at 50 nM for 24 h, then treated by 5 µM sodium arsenite for 5 h. The siRNA against MAPK was purchased from Qiagen. The sense sequence: UGCUGACUCCAAAGCUCUGdTdT ; the antisense sequence: CAGAGCUUUGGAGUCAGCAdTdT . The On-Target plus Smartpool siVEGF was from Dharmacon (Thermo Scientific, Pittsburgh, PA). The four target sequences are: GCAGAAUCAUCACGAAGUG; CAACAAAUGUGAAUGCAGA; GGAGUACCCUGAUGAGAUC; GAUCAAACCUCACCAAGGC. The cells were used for angiogenesis assay. After 96 h of incubation, the tumor plugs were trimmed off the CAM, and analyzed for angiogenesis response. The number of blood vessels as the index of angiogenesis was obtained by counting the branching of blood vessels in 2 square millimeter, which was normalized to that of the control group.

### Immunoblotting

BEAS-2B cells were starved for 24 h, then treated with sodium arsenite for 2 h (for p-AKT, AKT, p-ERK1/2 and ERK2 expression) or 6 h (for HIF-1α and HIF-1β expression). To study the effects of inhibitors, starved BEAS-2B cells were pre-treated with diphenylene iodonium (DPI), catalase, LY294002, or U0126 for 30 min, then treated with 5 µM of sodium arsenite as above. Total proteins were extracted from the cells using modified RIPA buffer supplemented with protein inhibitors, separated by SDS-PAGE, and blotted onto nitrocellular membrane. After blocking with 5% nonfat milk for 2 h, the membranes were incubated with the primary antibodies against HIF-1α, HIF-1β (BD, Franklin Lakes, NJ), phospho-AKT, phospho-ERK1/2, AKT (Cell Signaling Technology, Beverly, MA ), or ERK2 (Santa Cruz Biotechnology, Santa Cruz, CA) overnight. The specific protein signals were detected by incubation with horseradish peroxidase-conjugated antibodies and with a chemiluninescence reagent (Pierce, Rockford, IL) [27].

### Dichlorofluorescein diacetate (DCFH-DA) Staining for ROS

Cells were treated with sodium arsenite at different concentrations for 2 h, or treated with 5 µM sodium arsenite for different time points as indicated, then cells were incubated with DCFH- DA dye (Molecular Probe, Eugene, OR) for 15 min, washed with PBS buffer, and fixed with10% formaldehyde solution. The images were taken using a fluorescent microscope and analyzed as we previously described [26], [27].

### Luciferase Assay

Cells were seeded in 12-well plate at 60% confluence and cultured overnight. Cells were co-transfected with VEGF reporter and β-galactosidase (β-gal) plasmids and cultured for 15 h. Sodium arsenite at 0, 2.5, and 5 µM was added to the cells for 24 h. Luciferase assay was analyzed using luciferase assay system (Promega, San Luis Obispo, CA). The activity of β-gal was used as internal control of transfection efficiency. The relative luciferase (luc) activity was calculated as the ratio of luciferase/β-gal, and normalized to the control group [27].

### RT-PCR

Total RNAs were extracted using Trizol reagent (Invitrogen, Carlsbad, CA). Primers used for detecting VEGF and GAPDH are as follows:

VEGF forward primer, 5′-TCGGGCCTCCGAAACCATGA-3′;

VEGF reverse primer, 5′-CCTGGTGAGAGATCTGGTTC-3′;

GAPDH forward primer, 5′- CCACCCATGGCAAATTCCATGGCA-3′;

GAPDH reverse primer, 5′-TCTAGACGGCAGGTCAGGTCCACC-3′.

The amplification of VEGF was performed by PCR as previously described [27]. The expression levels of GAPDH were used as an internal control.

### Statistical Analysis

All values in this study were reported as mean ± SD. Student's unpaired t test was used for statistical analyses. The values were considered as significant difference at *p*<0.05.

## Results

### Arsenic treatment induces angiogenesis

**Figure 1:**
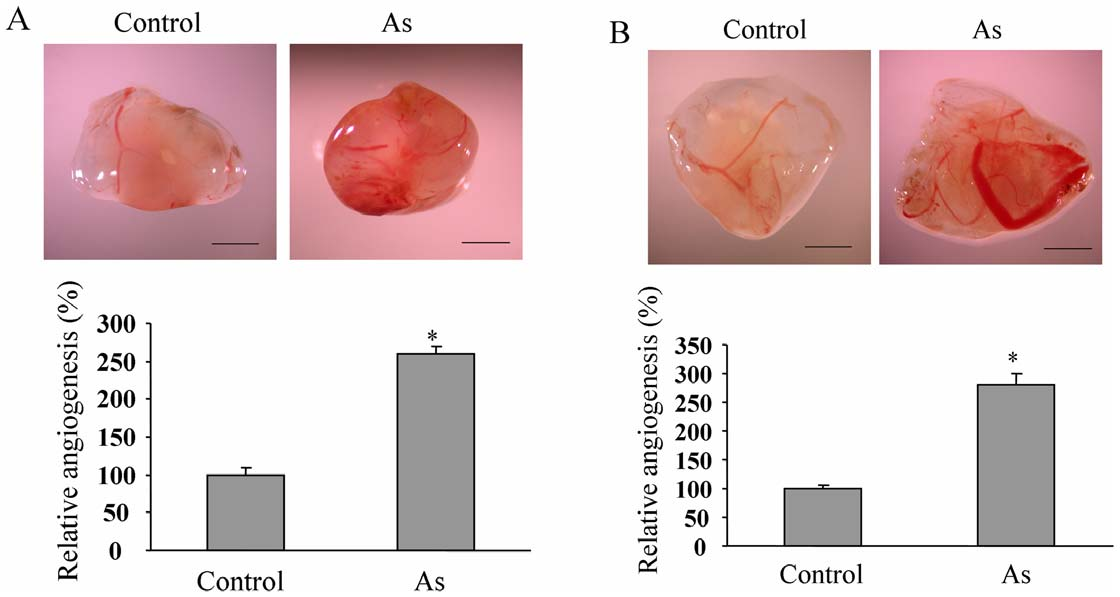
Arsenic induced angiogenesis. (A) A549 cells were treated without or with 5 µM of arsenic (As) for 5 h, then trypsinized, resuspended in serum-free medium (3×10^7^cells/ml, 0.1 ml), and mixed in 1∶1 ratio with Matrigel (Collaborative Biomedical Products, Bedford, MA). Aliquots of the mixture were then implanted onto the CAM of 9-day-old embryos. After 96 h incubation, the area around the implanted Matrigel was photographed with a Nikon digital camera. Bar: 2 mm (upper panel). The number of blood vessels was obtained by counting the branching of blood vessels, and the relative angiogenesis was obtained by normalizing to that of the control without arsenic treatment. The data represent the mean ± SD of the relative angiogenesis from eight different embryos (bottom panel). *, indicates that the relative angiogenesis index significantly increased in arsenic treatment group when compared with control group, *P*<0.05. (B) BEAS-2B cells were treated with or without arsenic to perform tumor angiogenesis assay as above. Bar: 2 mm. *, indicates that the relative angiogenesis index significantly increased in arsenic treatment group when compared with control group, *P*<0.05.

Angiogenesis is a vital pathological process during tumorigenesis. To investigate whether short time treatment of arsenic has effect on angiogenesis, human lung adenocarcinoma cell line A549 and immortalized normal human lung epithelial cells BEAS-2B were treated with 5 µM of sodium arsenite for 5 h. Angiogenesis assay was performed using the cells on the CAM model. Arsenic treatment strongly increased A549 cell-inducing angiogenesis to 2.5-fold when compared to the untreated control cells (Fig. 1A). Similarly, arsenic treatment also promoted BEAS-2B cells to induce angiogenesis by more than 2-fold (Fig. 1B). These results suggest that arsenic treatment induces angiogenesis in both immortalized lung epithelial cells and lung cancer cells.

### Arsenic activates AKT and ERK1/2 signaling pathways, and increases the expression of HIF-1α and VEGF

**Figure 2:**
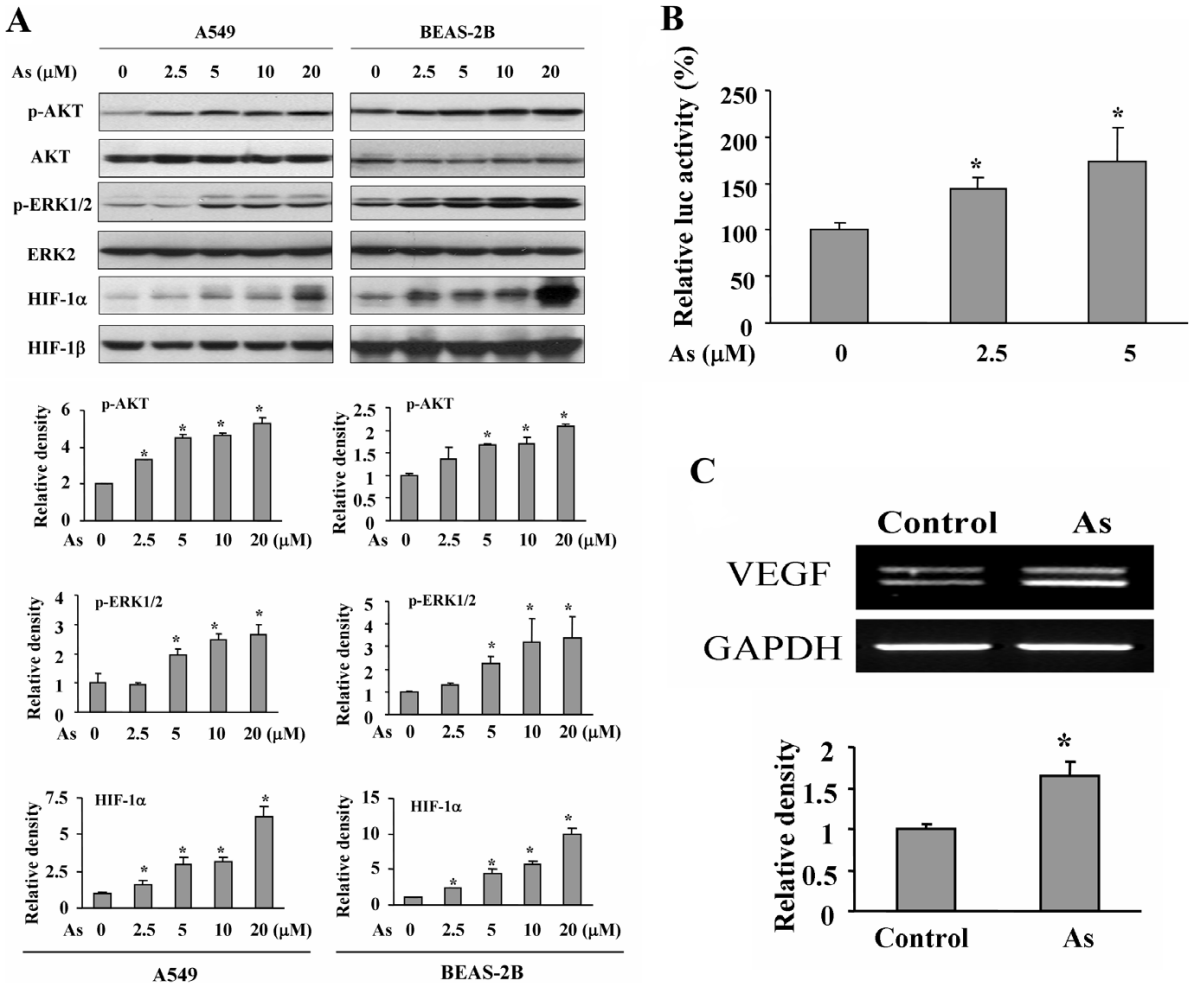
Arsenic treatment induced phospho-AKT and phospho-ERK1/2 activation, and increased HIF-1α and VEGF expression. (A) A549 and BEAS-2B cells were treated with different doses of arsenic (As) for 6 h, total proteins are subjected to Western blotting for HIF-1α and HIF-1β expression. A549 and BEAS-2B cells were cultured in serum-free medium for 24 h, then treated with different doses of arsenic for 2 h. Total proteins were subjected to Western blotting analysis for the levels of phospho-AKT, total AKT, phospho-ERK1/2, and ERK2 expression (upper panel). Relative densities of p-AKT, p-ERK1/2 and HIF-1α were analyzed by the ratio of p-AKT/AKT, p-ERK1/2/ERK2 and HIF-1α/HIF-1β using ImageJ software and normalized to those of control cells. The data represents the mean± SD from duplicate experiments (bottom panel). *, indicates significant increase when compared with the control cells, *P*<0.05. (B) BEAS-2B cells were seeded in 12-well plate. Cells were co-transfected with VEGF reporter and β-galactosidase (β-gal) plasmids and cultured for 15 h. Arsenic at 0, 2.5, and 5 µM was added for 24 h. Luciferase assay was performed by using luciferase assay system. The activity of β-gal was used as internal control of transfection efficiency. The relative luciferase activity was calculated as the ratio of luciferase/β-gal activity, and normalized to the control group. *, indicates that the relative luc activity significantly increased in arsenic treatment group when compared with the control group, *P*<0.05. *C,* BEAS-2B cells were treated without or with 5 µM of arsenic for 24 h. Total RNAs were extracted by Trizol and subjected to RT-PCR analysis of VEGF and GAPDH expression.

AKT (also known as Protein kinase B) is a serine/threonine kinase. The other important parallel signaling pathway is extracellular signal-regulated kinase (ERK). We showed that arsenic at 5 µM increased AKT and ERK1/2 activation in both A549 and BEAS-2B cells (Fig. 2A), suggesting that arsenic can activate these two signaling pathways in lung epithelial and cancer cells. To analyze the downstream effector of AKT and ERK1/2, we tested whether the expression of HIF-1 was induced by arsenic. HIF-1 is a heterodimeric transcription factor that is composed of a constitutive-expressing HIF-1β subunit and an oxygen-sensitive HIF-1α subunit [28]. A549 cells and BEAS-2B cells were treated with different doses of arsenic. As shown in Fig. 2A, arsenic treatment increased HIF-1α expression levels in both A549 and BEAS-2B cells. VEGF is a potent angiogenesis inducer that is upregulated by HIF-1 through the binding of HIF-1 to its promoter [29]. Similarly, VEGF transcriptional activation was significantly increased by arsenic treatment (Fig. 2B), and VEGF mRNA level was induced by arsenic treatment (Fig. 2C), suggesting that arsenic induces VEGF expression at transcriptional level.

### AKT and ERK1/2 are required for arsenic-inducing angiogenesis

**Figure 3:**
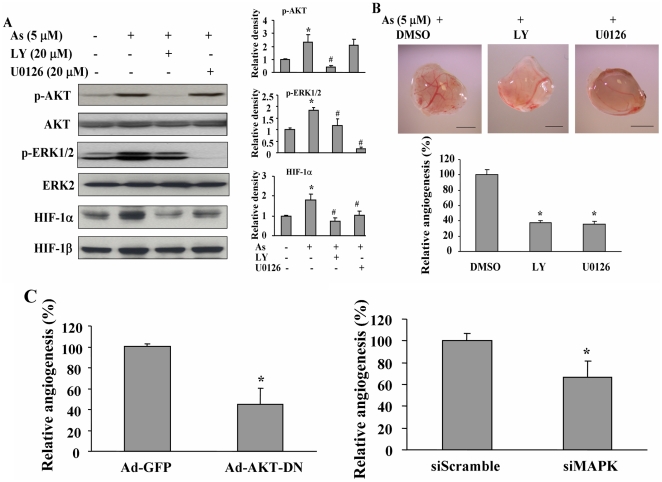
AKT and ERK1/2 pathways are required for arsenic-inducing HIF-1α expression and angiogenesis. (A) BEAS-2B cells were cultured in serum-free medium for 24 h, then cells were pre-treated with LY294002 or U0126 at 20 µM for 30 min. Arsenic at 5 µM was added to the cells for 2 h (for p-AKT, AKT, p-ERK1/2, and ERK2 expression) and 6 h (for HIF-1α and HIF-1β expression), respectively. Total proteins were analyzed by Western blotting to detect the expression of proteins as indicated (left panel). Relative densities of p-AKT, p-ERK1/2 and HIF-1α were analyzed as Fig. 2A (right panel). *, indicates significant increase when compared with the control cells, *P*<0.05. . #, indicates significant decrease when compared with the sodium arsenite treatment, *P*<0.05. (B) BEAS-2B cells were treated with 5 µM of arsenic. Cells were trypsinized, mixed with equal volume of Matrigel with or without 15 µM of LY294002 or U0126. Equal volume of DMSO was added as a negative control. Angiogenesis assay was performed as described in Fig. 1. *, indicates that the relative angiogenesis index was significantly decreased when compared with DMSO control group, *P*<0.05. (C) BEAS-2B cells were infected with adenovirus carrying GFP (Ad-GFP) or AKT dominant negative (Ad-AKT-DN) at 20 MOI (upper panel), or transfected with scrambled control of siRNA (siScramble) or siMAPK at 50 nM (bottom panel). After 24 h, cells were treated with arsenic and angiogenesis assay was performed as above. *, indicates that the relative angiogenesis index was significantly decreased when compared with Ad-GFP or siScramble control group, *P*<0.05.

Pre-treatment of BEAS-2B cells with PI3K and MAP kinase inhibitors LY294002 and U0126 blocked the activation of phospho-AKT or phospho-ERK induced by arsenic, and decreased arsenic-inducing HIF-1α but not HIF-1β protein levels (Fig. 3A). This result suggests that both AKT and ERK1/2 are required for arsenic-induced HIF-1 expression. To test whether AKT and ERK are required for arsenic-inducing angiogenesis, BEAS-2B cells were treated by LY294002 or U0126, and used to do angiogenesis assay. We found that LY294002 or U0126 treatment inhibited angiogenesis by more than 50% when compared to the control (Fig. 3B). To further specifically inhibit AKT or ERK, the cells were infected with adenoviruses carrying GFP (Ad-GFP) and AKT dominant negative (Ad-AKT-DN) to specifically inhibit AKT activation; or transfected with scrambled control siRNA and siMAPK to knockdown MAPK expression. These cells were used to perform angiogenesis assay. Similar to the effect of LY294002 and U0126 treatment, BEAS-2B cells treated with Ad-AKT-DN or siMAPK showed significant inhibition of angiogenesis responses with the reduction to 50% and 70% as compared to those of the Ad-GFP or siScrambled control group, respectively (Fig. 3C). These results further confirm that AKT and ERK1/2 are required for arsenic-inducing angiogenesis.

### Arsenic induces reactive oxygen species (ROS) generation, which is necessary for arsenic-inducing angiogenesis

**Figure 4:**
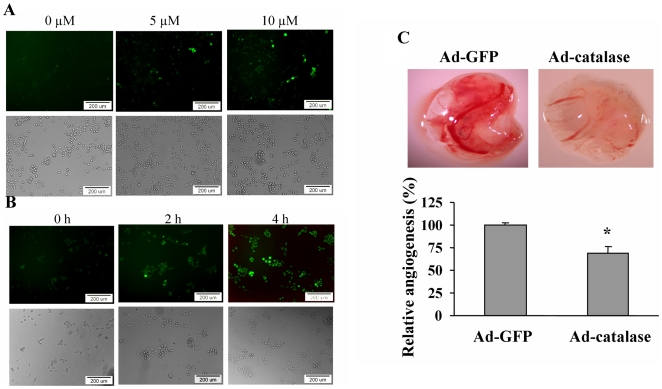
Arsenic induced ROS production in BEAS-2B cells, which was required for angiogenesis. (A) BEAS-2B cells were seeded into 6-well plates. Cells were treated with different doses of arsenic as indicated in serum-free medium. DCFH-DA at 5 µM was added to the cells for 15 min. Then the cells were washed and fixed, and the fluorescent images were captured using a fluorescent microscope (upper panel). The corresponding phase micrographs were shown in the bottom panel. (B) BEAS-2B cells were seeded into the 6-well plate. The cells were then cultured in serum-free medium with arsenic at 5 µM for different time points as indicated. DCFH-DA staining was performed as above. (C) BEAS-2B cells were infected with adenovirus carrying GFP (Ad-GFP) and catalase (Ad-catalase), respectively at 20 MOI. After 24 h, cells were treated with 5 µM arsenic for 5 h to perform angiogenesis assay. *, indicates that the relative angiogenesis index was significantly decreased when compared with Ad-GFP control group, *P*<0.05.

Previous study showed that low level of sodium arsenic at 100 nM exposure for long time renders human keratinocytes apoptotic resistant and induces ROS generation [30]. To study whether short time exposure of arsenic may induce ROS levels, BEAS-2B cells were treated with different doses of arsenic for 2 h, or treated with 5 µM arsenic for 2 h and 4 h. As shown in Fig. 4A, ROS production levels were higher with arsenic treatment at 10 µM than at 5 µM, and were higher with arsenic treatment at 5 µM for 4 h than for 2 h (Fig. 4B). These results demonstrate that ROS are induced by arsenic treatment.

To further test whether arsenic-inducing ROS are required for angiogenesis, BEAS-2B cells were infected with adenovirus carrying catalase, a scavenger of hydrogen peroxide, or GFP (as control) at 20 MOI. These cells were treated by arsenic and used for angiogenesis assay as previously described. Overexpression of catalase in arsenic-treated BEAS-2B cells significantly decreased angiogenesis when compared to the control group (Fig. 4C). These results indicate that arsenic induces ROS production which is required for arsenic-inducing angiogenesis.

### ROS induction is required for arsenic-induced AKT and ERK1/2 activation and HIF-1 expression; and HIF-1 elevation is required for arsenic-induced angiogenesis

**Figure 5:**
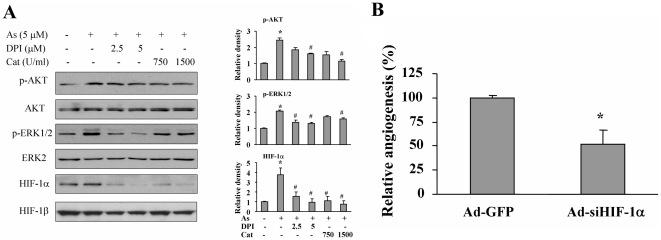
ROS are required for AKT and ERK1/2 activation, HIF-1α expression, and angiogenesis. (A) BEAS-2B cells were cultured in serum-free medium for 24 h, then cells were pre-treated with DPI or catalase for 30 min. Arsenic at 5 µM was added to the cells for 2 h or 6 h as above. The specific proteins are analyzed by Western blotting (left panel). The relative densities of p-AKT, p-ERK1/2 and HIF-1α were determined as above. *, indicates significant increase when compared with the control cells, *P*<0.05. . #, indicates significant decrease when compared with the sodium arsenite treatment alone, *P*<0.05. (B) BEAS-2B cells were infected with adenovirus carrying GFP and HIF-1α siRNA (Ad-GFP and Ad-siHIF-1α, respectively) at 20 MOI for 24 h, then the cells were treated with 5 µM arsenic for 5 h and angiogenesis assay was performed as above. *, indicates that the relative angiogenesis index was significantly decreased when compared with the control group, *P*<0.05.

To explore the molecular mechanism of ROS in mediating angiogenesis, we showed that ROS inhibitors DPI and catalase significantly suppressed AKT and ERK1/2 activation (Fig. 5A). Similarly, the expression of HIF-1α was decreased by ROS inhibitors (Fig. 5A). To determine whether HIF-1α elevation is required for arsenic-induced angiogenesis, BEAS-2B cells were transduced by adenovirus carrying siHIF-1α or GFP. These cells were treated by arsenic to perform angiogenesis assay as above. As shown in Fig. 5B, HIF-1α knockdown markedly inhibited the angiogenesis with the reduction of angiogenesis responses by 50%. These results suggest that arsenic induces HIF-1 expression through ROS generation, AKT and ERK1/2 activation, and HIF-1 elevation is required for arsenic-induced angiogenesis.

### ROS production is required for arsenic-induced VEGF expression, and VEGF is required for arsenic-induced angiogenesis

**Figure 6:**
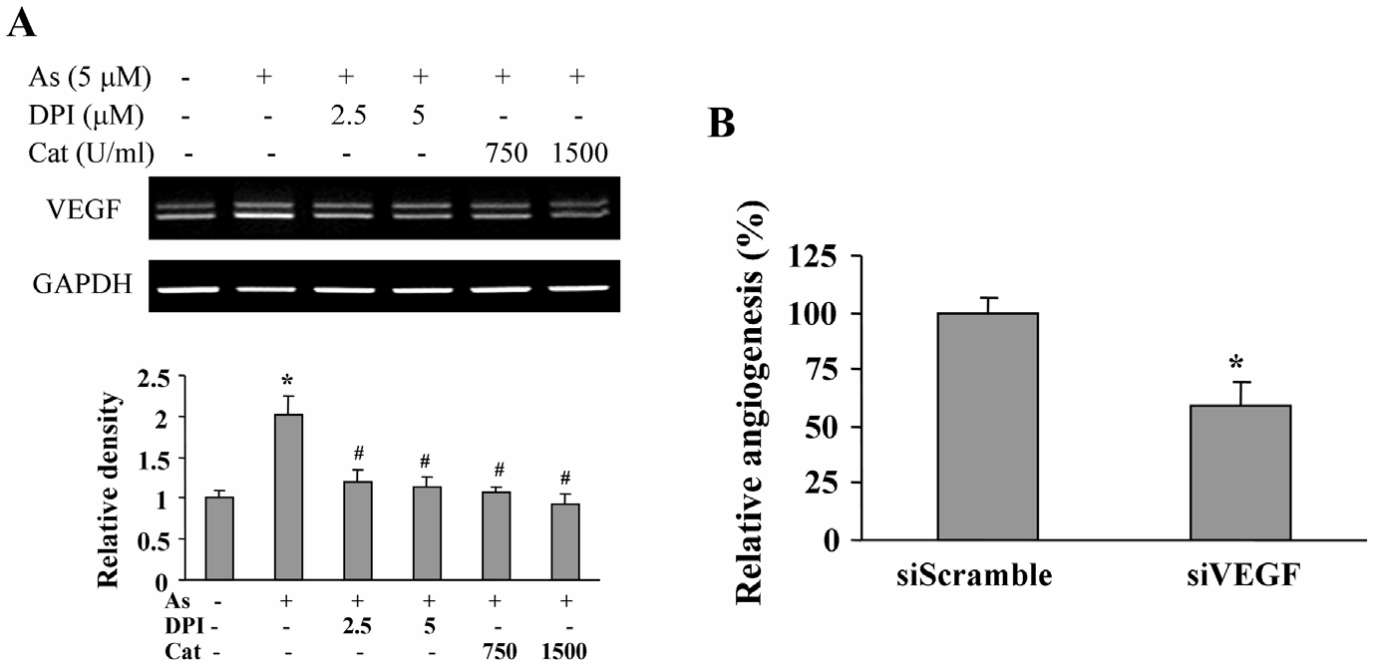
VEGF is required for arsenic-inducing angiogenesis. (A) BEAS-2B cells were treated with DPI or catalase for 30 min, then with 5 µM arsenic for 24 h. Total RNAs were extracted by Trizol, and analyzed by RT-PCR for VEGF and GAPDH expression (upper panel). Relative density of VEGF was analyzed by the ratio of VEGF/GAPDH using ImageJ software and normalized to control cells. The data represents the mean± SD from duplicate experiments (bottom panel). *, indicates significant increase when compared with the control cells, *P*<0.05. . #, indicates significant decrease when compared with the sodium arsenite treatment alone, *P*<0.05. (B) BEAS-2B cells were transfected with VEGF siRNA and scrambled siRNA (siVEGF and siScramble, respectively). After the transfection for 24 h, cells were treated with 5 µM arsenic for 5 h to perform angiogenesis assay. *, indicates that the relative angiogenesis index was significantly decreased in siVEGF treatment group when compared with siScramble group, *P*<0.05.

It is known that HIF-1 induces VEGF transcription by the binding of HIF-1 to VEGF promoter [29]. Both HIF-1 and VEGF play a pivotal role in regulating anigogenesis [17]. To determine whether ROS are required for arsenic-induced VEGF expression, the cells were pretreated with DPI and catalase, followed by the arsenic treatment. DPI and catalase suppressed arsenic-induced VEGF expression (Fig. 6A). To assess whether VEGF expression is required for arsenic-induced angiogenesis, the cells were transfected with VEGF siRNA or scrambled siRNA control, followed by the treatment of arsenic. These treated cells were used to perform angiogenesis assay. Similar to the results of HIF-1α knockdown, VEGF knockdown also decreased arsenic-induced angiogenesis by 50% (Fig. 6B), suggesting that arsenic can increase HIF-1 expression, which in turn regulates VEGF expression for inducing angiogenesis.

## Discussion

Environmental exposure to arsenic is commonly in the forms of either arsenite (As^+3^) or arsenate (As^+5^), which is widely present in environments such as water, air and soil. Arsenite is the predominant form of arsenic in contaminated water and is the form mainly attributed to the induction of human cancers, including lung, skin, bladder, and liver cancer. People are exposed to arsenic through the arsenic-containing drinking water, food, and air-dust. The occupational exposure to arsenic may be through the inhalation of arsenic dusts in the production and distribution process. There are more than 1.5 million industrial workers that are potentially exposed to arsenic and arsenic compounds during the product manufacturing and distribution according to the NIOSH estimate [31]. Lung is one of the major organs to arsenic exposure. As a metalloid carcinogen, arsenic is known to be associated with higher incidence of both lung cancer and non-malignant pulmonary diseases. In this study, we used sodium arsenic to treat both human lung adenocarcinoma cells A549 and immortalized human lung epithelial cells, BEAS-2B; and demonstrated that short time exposure of arsenic in lung epithelial cells induced angiogenesis, suggesting that arsenic-inducing angiogenesis is involved in arsenic-inducing lung carcinogenesis.

ROS include superoxide, hydrogen peroxide and hydroxyl radical. Previous studies demonstrated that arsenic induced ROS production and DNA damage [4], [32]-[34]. We found that arsenic induced ROS production in a dose-dependent manner, and that suppression of ROS generation by catalase inhibited arsenic-inducing angiogenesis, suggesting that ROS production is required for arsenic-inducing angiogenesis.

In this study, we showed that arsenic treatment led to the induction of VEGF and HIF-1 expression. HIF-1 composes of the heterodimers of HIF-1α and HIF-1β subunits, which regulates VEGF expression at transcriptional level. VEGF is a key angiogenesis factor for stimulating endothelial cell growth and angiogenesis [35], [36]. Inhibition of VEGF-inducing angiogenesis greatly decreased tumor growth *in vivo* [37]. In chronic pulmonary diseases such as COPD, pulmonary endothelial dysfunction and change of levels of VEGF and its receptor were found to be involved in the development of vascular disease [38], [39]. HIF-1 also regulates many other target genes that are involved in crucial aspects of cancer biology including angiogenesis [28]. Overexpression of HIF-1 is a hallmark of many kinds of solid tumors, and is also associated with skeletal muscle disease COPD [28], [40], [41]. Recent study showed that chronic exposure to low-dose As^+3^ stimulated tumor growth through induction of angiogenesis [42]. This study showed that the knockdown of HIF-1 and VEGF using siRNAs against HIF-1α or VEGF inhibited arsenic-inducing angiogenesis, demonstrating the important role of HIF-1α and VEGF in arsenic-inducing angiogenesis.

To further determine the signaling pathways in upregulating HIF-1 and VEGF expression, we found that AKT and ERK1/2 were activated by arsenic, and that inhibition of AKT or ERK1/2 by their chemical inhibitors, dominant negative molecule, or siRNAs blocked arsenic-inducing angiogenesis. These findings suggest that AKT and ERK pathways are involved in arsenic-stimulating angiogenesis in lung epithelial cells, which is consistent with previous study showing that arsenic induced ROS generation and ERK activation [4]. This study reveals the important biological function of the previous observation by our lab and others that showed AKT and ERK are upstream regulators of HIF-1 and VEGF [27], [43]–[45]. We further demonstrated that inhibition of ROS generation blocked HIF-1 and VEGF induction, AKT and ERK1/2 activation, and angiogenesis in response to arsenic treatment. This new result provides an important link from ROS, signaling molecules, to angiogenesis. In addition to acute treatment of arsenic, we found that chronic treatment with sodium arsenite had similar effects.

Taken together, this study demonstrated that acute treatment of arsenic in lung epithelial cells induced angiogenesis, arsenic increases ROS induction for inducing angiogenesis, and ROS transmits the angiogenesis signals to downstream targets AKT, ERK1/2, HIF-1, and VEGF. This study shows a novel molecular mechanism of arsenic in affecting human lung epithelial cells to induce angiogenesis. This finding may provide useful information for developing mechanism-based prevention and treatment of lung cancer induced by arsenic in the future.
